# Effects of Tannic Acid on Lipid and Protein Oxidation, Color, and Volatiles of Raw and Cooked Chicken Breast Meat during Storage

**DOI:** 10.3390/antiox5020019

**Published:** 2016-06-13

**Authors:** Marwan Al-Hijazeen, Eun Joo Lee, Aubrey Mendonca, Dong Uk Ahn

**Affiliations:** 1 Department of Animal Science, Iowa State University, Ames, IA 50011, USA; marwana@mutah.edu.jo (M.A.-H.); duahn@iastate.edu (D.U.A.); 2 Department of Food and Nutrition, University of Wisconsin-Stout, Menomonie, WI 54751, USA; 3 Department of Food Science and Human Nutrition, Iowa State University, Ames, IA 50011, USA; amendon@iastate.edu

**Keywords:** chicken breast meat, tannic acid, lipid oxidation, protein oxidation, volatiles, color

## Abstract

The objective of this study was to determine the effect of tannic acid (TA) on the oxidative stability and the quality characteristics of ground chicken breast meat. Five treatments including (1) control (none added), (2) 2.5 ppm TA, (3) 5 ppm TA, (4) 10 ppm TA, and (5) 5 ppm butylated hydroxyanisole (BHA) were added to boneless, skinless ground chicken breast meat, and used for both raw and cooked meat studies. For the raw meat study, the ground chicken breast meat was packaged in oxygen-permeable bags and stored at 4 °C for 7 days. For the cooked study, raw ground meat samples were vacuum-packaged in oxygen-impermeable vacuum bags, cooked in-bag to the internal temperature of 75 °C, re-packaged in oxygen-permeable bags, and then stored. Both raw and cooked meats were analyzed for lipid and protein oxidation, color, and volatiles (cooked meat only) at 0, 3, and 7 days of storage. Raw meats with 10 ppm of TA added had significantly (*p* ≤ 0.05) lower lipid and protein oxidation than other treatments during storage. In addition, TA at 10 ppm level maintained the highest color a*- and L*-values during storage. Cooked chicken breast meat with 5 and 10 ppm TA added produced significantly (*p* ≤ 0.05) lower amounts of off-odor volatiles than other treatments. Among the volatile compounds, the amount of hexanal increased rapidly during storage for cooked meat. However, meats with 5 and 10 ppm TA added showed the lowest amount of hexanal and other aldehydes related to lipid oxidation, indicating a strong antioxidant effect of TA in cooked chicken breast meat. Furthermore, the differences in aldehydes among the treatments were bigger in cooked than in raw meat, indicating that the antioxidant effect of TA in cooked meat was greater than that in raw meat. Therefore, TA at >5 ppm can be used as a good natural preservative in cooked chicken meat to maintain its quality during storage.

## 1. Introduction

The use of synthetic antioxidants is discouraged due to their potential adverse effects on human health, and the poultry meat industry is actively seeking natural antioxidants to replace the synthetic ones. Several natural antioxidants from plants showed positive effects in improving meat quality and extending shelf life [1,2,3,4].

Meat color and odor are important attributes for the consumer evaluation on meat quality [5,6]. Usually, fresh meat is characterized by a cherry-red color due to the formation of oxymyoglobin (OxyMb). However, oxidation of the heme pigment will convert the red color to brown [7]. Lipid oxidation is considered as the major problem affecting meat quality [8] because it changes color, generates off-odor [9], and impairs protein functionality [10]. Protein functionality such as solubility, emulsification, water binding capacity, and texture are also affected by lipid oxidation and their interactions with protein oxidation products [10,11].

The progress of oxidation in fresh meat depends on many internal factors such as catalyst (Iron Fe, Cu, *etc.*), antioxidant capacity, pH, fatty acid composition, and free radical formation. However, many external factors such as high storage temperature, oxygen availability, meat processing method, and additives also can influence lipid and protein oxidation in meat [12]. Therefore, finding new natural antioxidants to resolve these problems in meat and other foods is important.

Herbs and plant extracts such as rosemary [13], oregano [14], grape seed and bearberry extracts [15,16], plum extract [17], and garlic and onion oleoresins [8] have been tested to prevent lipid oxidation in meat because they contained high levels of antioxidants [4]. Plants produce a wide variety of “secondary compounds” including alkaloids, terpenes, and phenolics in their cells to protect themselves from external herbivores and diseases [18]. Among these compounds, tannins have metal-chelating, antioxidant, and protein precipitating activities [19], and show positive effects on meat color stability and extend their self-life [20].

The tannins are defined as water-soluble phenolic compounds with molecular weights between 500 and 3000 Da, and have special properties such as precipitating alkaloids, gelatin, and other proteins [21]. These properties of tannins are based on their chemical structures, which have two or three phenolic hydroxyl groups on the phenyl ring (polyphenol). Tannins are classified into two different main groups—hydrolysable and condensed tannins. The hydrolyzable type of tannins contains polyhydric alcohol and hydroxyl groups, which are esterified by gallic acid (gallotannins) or to hexahydroxy diphenic acid (ellagitannins) [22]. Condensed tannins are more complex in their structure compared to hydrolysable tannins, and are mainly produced by two main polymerized products of flavan-3-ols and flavan-3,4-diols, or a mixture of both [23].

Tannic acid is a yellowish-white to light-brown powder, and is soluble in water and alcohol (hydrolysable tannins). The antioxidant activity of tannic acid was explained by several researchers through their ability to prevent hydroxyl radical formation [24], metal-chelating activity [22], and radical-scavenging activity [25]. These activities make the tannic acid a possible replacement for synthetic antioxidants [26,27]. Tannic acid can be obtained in two different ways: (1) solvent extraction of nutgalls or excrescences from young twigs of Quercus infectoria Oliver, and (2) solvent extraction of the seed pod of Tara (Caesalpinia spinosa), or the nutgalls of various sumac species, including Rhus semialata, R. coriaria, R. galabra, and R. typhia [28]. The commercial formula for tannic acid is given as C_76_H_52_O_46_ and is composed of a mixture of polygalloyl glucose and polygalloyl quinnic acid ester depending on the plant extract sources and purification methods used [18,19]. Tannic acid is classified as generally recognized as safe (GRAS) by the Food and Drug Administration (FDA), and up to 10 ppm of tannic acid is allowed to be used in meat products [28]. However, little work has been done to determine their effect on the storage stability and quality characteristics of ground chicken meat.

The objective of this study was to investigate the effect of adding tannic acid on the stability and quality of ground chicken meat (raw and cooked meat) during storage.

## 2. Materials and Methods

Tannic acid powder containing 90% tannin was obtained from Sigma-Aldrich (St. Louis, MO, USA). The tannic acid contained gallic acid, monogalloyl glucose, digalloyl glucose, trigalloyl glucose, tetragalloyl glucose, pentagalloyl glucose, ESA galloyl glucose, EPTA galloyl glucose, and octagalloyl glucose. The product is hydrolyzable tannin obtained from oak gall nuts from *Quercus infectoria.*

### 2.1. Sample Preparation

One hundred and twenty, 6-week-old broilers raised on a corn-soybean meal diet were slaughtered using the USDA guidelines [29]. The chicken carcasses were chilled in ice water for 2 h and drained in a cold room, and the breast muscles were separated from the carcasses 24 h after slaughter. The breast muscles were ground twice a through a 10-mm and a 3-mm plates (Kitchen Aid, Inc., St. Joseph, MI, USA) after removing skins before use. Five treatments including (1) control (none added), (2) 2.5 ppm tannic acid, (3) 5 ppm tannic acid, (4) 10 ppm tannic acid, and (5) 5 ppm butylated hydroxyanisole (BHA) were prepared. Tannic acid (0.1 g) was dissolved in 50 mL of de-ionized distilled water (DDW) and stored in a dark area to prevent exposure to light. BHA powder (0.1 g) was dissolved in 10 mL of 100% ethanol, and then mixed with 50 mL mineral oil to make a stock solution. The ethanol added was removed using a rotary evaporator (BUCH Rotavapor, Model R-200, BUCHI Co., New Castle, DE, USA) at (70 °C, 175 mbar vacuum pressure) before adding the stock solution to meat samples. Each additive treatment was added to the ground breast meat and then mixed for 2 min in a bowl mixer (Model KSM 90; Kitchen Aid Inc., St. Joseph, MI, USA). All treatments were added with the same amounts of mineral oil to provide the same conditions.

For raw-meat study, the prepared meat samples (approximately 100 g each) were individually packaged in oxygen-permeable bags (polyethylene, 4” × 6”, 2 mil, Associated Bag Co., Milwaukee, WI, USA), stored at 4 ℃ for 7 days, and analyzed for lipid and protein oxidation, and color at 0, 3, and 7 days of storage.

The same preparation method was used for cooked meat study, but the raw meat samples were vacuum packaged in oxygen-impermeable vacuum bags (O_2_ permeability, 9.3 mL O_2_/m^2^/24 h at 0 °C, Koch, Kansas City, MO, USA), and the meats were cooked in-bag in a 90 °C water bath (Isotemp^®^, Fisher Scientific Inc., Pittsburgh, PA, USA) until the internal temperature of the meat reached to 75 °C. After cooling to room temperature, the cooked meat was individually re-packaged in oxygen-permeable bag (polyethylene, 4” × 6”, 2 mil, Associated Bag Co., Milwaukee, WI, USA), stored at 4 °C for 7 days, and analyzed for lipid and protein oxidation and volatiles at 0, 3, and 7 days of storage.

### 2.2. 2-Thiobarbituric Acid-Reactive Substances (TBARS) Measurement

Lipid oxidation was determined using a TBARS method [30]. Five grams of ground chicken meat were weighed into a 50-mL test tube, added with 50 μL BHT (7.2%) and 15 mL of deionized distilled water (DDW), and homogenized using a Polytron (Type PT 10/35, Brinkman Instruments Inc., Westbury, NY, USA) for 15 s at high speed. One milliliter of the meat homogenate was transferred to a disposable test tube (13 × 100 mm), and thiobarbituric acid/trichloroacetic acid solution (15 mM TBA/15% TCA, 2 mL) was added. The mixture was vortex-mixed and incubated in a boiling water bath for 15 min to develop color. Then, samples were cooled in ice-water for 10 min, mixed again, and centrifuged for 15 min at 2500× *g* at 4 °C. The absorbance of the resulting supernatant solution was determined at 532 nm against a blank containing 1 mL of DDW and 2 mL of TBA/TCA solution. The amounts of TBARS were expressed as mg of malondialdehyde (MDA) per kg of meat.

### 2.3. Color Measurement

The color of meat was measured on the surface of meat samples using a Konica Minolta Color Meter (CR-410, Konioka Minolta, Osaka, Japan). The color meter was calibrated using an illuminant source C (average daylight) on a standard white ceramic tile covered with the same packaging film as the ones used for meat samples to negate the color and light reflectance properties of the packaging material. The color was expressed as CIE L*- (lightness), a*- (redness), and b*- (yellowness) values [31]. The areas selected for color measurement were free from obvious defects that may affect the uniform color readings. An average of two random readings on the top of the sample surface was used for statistical analysis.

### 2.4. Volatile Analysis

Volatiles of samples were analyzed using a Solatek-72 Multimatrix-Vial Auto-sampler/Sample Concentrator 3100 (Tekmar-Dohrmann, Cincinnati, OH, USA) connected to a GC/MS (Model 6890/5973; Hewlett-Packard Co., Wilmington, DE, USA) according to the method of Ahn *et al.* [32]. Sample (3 g for raw meat and 2 g for cooked meat) was placed in a 40-mL sample vial, flushed with helium gas (40 psi) for 3 s, and then capped airtight with a Teflon-fluorocarbon resin/silicone septum (I-Chem Co., New Castle, DE, USA). Samples from different treatments were randomly organized on the refrigerated (4 °C) holding trying to minimize the oxidative changes among treatments during analysis. The meat sample was purged with helium (40 mL/min) for 14 min at 20 °C. Volatiles were trapped using a Tenax/charcoal/silica column (Tekmar-Dohrmann) and desorbed for 2 min at 225 °C, focused in a cryofocusing module (−70 °C), and then thermally desorbed into a capillary column for 2 min at 225 °C. An HP-624 column (7.5 m, 0.25 mm i.d., 1.4 μm nominal), an HP-1 column (52.5 m, 0.25 mm i.d., 0.25 μm nominal), and an HP-Wax column (7.5 m, 0.250 mm i.d., 0.25 μm nominal) were connected using zero dead-volume column connectors (J &W Scientific, Folsom, CA, USA). Ramped oven temperature was used to improve volatile separation. The initial oven temperature of 25 °C was held for 5 min. After that, the oven temperature was increased to 85 °C at 40 °C per min, increased to 165 °C at 20 °C per min, and then increased to 230 °C at 5 °C per min and held for 2.5 min at the temperature. Constant column pressure at 22.5 psi was maintained. The ionization potential of MS was 70 eV, and the scan range was 20.1–350 m/z. The identification of volatiles was achieved by the Wiley Library (Hewlett-Packard Co., Wilmington, DE, USA). The area of each peak was integrated using ChemStation™ software (Hewlett-Packard Co., Wilmington, DE, USA), and the total peak area (total ion counts × 10^4^) was reported as an indicator of volatiles generated from the samples.

### 2.5. Protein Oxidation (Total Carbonyl)

Protein oxidation was determined by the method by Lund *et al.* [33] with minor modifications. One gram of meat sample was added to 10 mL of pyrophosphate buffer (2.0 mM Na_4_P_2_O_7_, 10 mM Trizma-maleate), 100 mM KCL, 2.0 mM MgCl_2_, and 2.0 mM ethylene glycol tetraacetic acid, pH 7.4) and homogenized using a Brinkman Polytron (Type PT 10/35). Two equal amounts of meat homogenate (2 mL) were taken from a sample, precipitated with 2 mL of 20% trichloroacetic acid, and centrifuged at 12,000× *g* for 5 min at room temperature. After centrifugation, one of the pellets was dissolved with 2 mL of 10 mM 2,4-dinitrophenylhydrazine in 2 M HCl and the other one was dissolved with 2 M HCl (blank), and were incubated for 30 min in the dark. During the incubation, the samples were vortex-mixed for 10 s every 3 min. The protein was further precipitated with 2 mL of 20% trichloroacetic acid and centrifuged at 12,000× *g* for 5 min. The 2,4-dinitrophenylhydrazine was removed by washing the pellets 3 times with 4 mL of 10 mM HCl in 1:1 (vol/vol) ethanol:ethyl acetate, followed by centrifuging at 12,000× *g* for 5 min. The pellets were finally solubilized in 2 mL of 6.0 mM guanidine hydrochloride dissolved in 20 mM potassium dihydrogen phosphate (pH = 2.3). The samples were kept at 5 °C overnight and centrifuged to remove insoluble materials. The absorbance of supernatants was read at 370 nm and the value of blank sample was subtracted from their corresponding sample value. The protein concentration of meat samples was measured using Protein Assay Kit (Bio-Rad Laboratories, Hercules, CA, USA) following Microplate Assay protocol at 280 nm (BioTek-Gen5 Microplate data collection & analysis software/BioTek Instruments, Inc., Model S4MLFPTA, Winooski, VT, USA). The carbonyl content was calculated as nmol/mg protein using absorption coefficient of 22,000/M/cm as described by Levine *et al.* [34].

### 2.6. Statistical Analysis

Data were analyzed using the procedures of the generalized linear model (Proc. GLM, SAS program, version 9.3, 2012, SAS Institute Inc., Cary, NC, USA) [35]. Mean values and standard error of the means (SEM) were reported. The significance was defined at *p* < 0.05 and Tukey or Tukey’s Multiple Range tests were used to determine whether there is a significant difference between the mean values.

## 3. Results and Discussion

### 3.1. Lipid Oxidation

In the raw chicken meat, there was no significant difference (*p* > 0.05) in TBARS between treatments at day 0. In addition, tannic acid at 2.5 ppm did not show any significant antioxidant effect on the chicken breast meat during storage. However, 5 ppm BHA or tannic acid at 5 and 10 ppm showed significant antioxidant effects after seven days of storage (Table 1). Tannic acid at 10 ppm showed the strongest antioxidant effects of all during storage. This result is in agreement with that of Maqsood and Benjakul [26] who found that tannic acid at 100–200 ppm effectively decreased both peroxide value and TBARS values in catfish slices. They found that tannic acid exhibited the highest antioxidant activities (peroxide value, conjugated diene, TBARS values) in both fish oil emulsion and fish mince among the phenolic compounds (catechin, caffeic acid, ferulic acid, and tannic acid) they had tested [27]. They explained that the high antioxidant effect of tannic acid was due to its ability to chelate non-heme iron in the fish mince. Iron is considered a strong pro-oxidant transitional metal that can increase lipid oxidation in meat. Because of their chemical structure, tannic acid inhibits hydroxyl radical formation from the Fenton reaction by complexing ferrous ions [24]. Lopes *et al.* [24] found that the antioxidant activity of tannic acid is mainly due to iron chelating rather than ^.^OH-scavenging activity. Maqsood and Benjakul [20] also reported that when tannic acid (200 mg/kg) was added to the refrigerated ground beef, its peroxide and TBARS values were significantly lower than that of the control.

Research using tannic acid in cooked meat products has been very scarce until recently. The initial TBARS values in cooked meat showed that all treatments added with tannic or BHA showed significant antioxidant effects (Table 1). The initial TBARS values of cooked meat were similar to those of the raw meat, but rapidly increased during storage, especially in control. Under oxygen permeable conditions, cooked meat oxidized very rapidly because of the denaturation of antioxidant enzymes and the structural damages in the membrane during cooking, which can expose phospholipids to the pro-oxidant environment [11,36]. Therefore, the difference in lipid oxidation between treatments in cooked meat became clearer than the raw meat. The greatest antioxidant effect was observed when 10 ppm tannic acid was added to the meat. Maqsood and Benjakul [20] also found that tannic acid can be a good additive that retarded the initiation and propagation steps of lipid oxidation reaction when added to the ground beef meat. In addition, they found that the tannic acid radical was characterized as stable and had low energy or low activity to initiate oxidation of unsaturated fatty acid which increased its effectiveness [27]. Tannic acid at 10 ppm had a stronger antioxidant effect than 5 ppm tannic acid and 5 ppm BHA at day 7 of storage (Table 1). This suggested that tannic acid could be a good replacement for synthetic antioxidant in foods.

### 3.2. Protein Oxidation

Protein and lipid oxidation are major concerns that affect meat quality during storage [37]. It has been reported that protein oxidation can lead to changes in overall properties of meat proteins such as gelation, viscosity, solubility, and emulsification [38]. Little information about the relationships between protein oxidation and their effect on some meat quality attributes is available. In this study, different levels of tannic acid were investigated to study their effect on the total carbonyl formation (nmol/mg of protein). There were no significant differences (*p* > 0.05) in the amount of carbonyl content among the tannic acid treatments in raw chicken breast meat during the first three days of storage (Table 2). The changes of protein oxidation values in raw chicken breast meat during the 7-day storage period were very low (0.54 at day zero to 0.82 at day 7 for control samples). This was in agreement with Xiao *et al.* [39] who found that the total carbonyl content in raw chicken meat patties stored aerobically at 4 ℃ increased from 0.46 to 0.81 nmol/mg of protein. Tannic acid at level 10 ppm was the only treatment that showed a significant antioxidant effect (*p* < 0.05) at day 7 of storage. Maqsood and Benjakul [20] found that tannic acid reduced the degradation of myosin heavy chain and actin in meat by suppressing microbial growth during storage. This indicated that the antimicrobial effect of tannic acid could have contributed to the lower total carbonyl formation in meat with different levels of tannic acid. On the other hand, the formation of secondary products (carbonyl and others) of protein oxidation is also related to the degree of lipid oxidation because lipid oxidation is directly related to protein oxidation [40].

The total carbonyl values of control cooked meat were higher than those of the raw meat after 3 days of storage and reached up to 2 nmol/mg protein after 7 days of storage (Table 2). Other researchers reported that total estimated carbonyl contents were in the range of 1–3 nmol/mg protein for raw meat and up to 5 nmol/mg protein for cooked meat products [41,42]. Adding tannic acid at 10 ppm successfully delayed the total carbonyl formation in cooked meat during storage. However, tannic acids at 5 and 10 ppm were more effective than other treatments in inhibiting carbonyl formation during storage. These results agreed well with the TBARS values of cooked ground chicken meat during storage. Understanding the mechanism of tannic acid in preventing protein oxidation still needs further investigation, but the iron-chelating activity of tannic acid could be a major reason for delaying total carbonyl formation in meat [24,43].

### 3.3. Color Values

Chicken breast meat contains much lower myoglobin content than meats from other animal species such as beef, goat, pork and sheep. Meat color is an important quality parameter that affects the consumer’s purchase decision [7]. However, little research was done on the color of ground chicken meat [44]. Table 3 showed that L*-values of meat decreased significantly (*p* < 0.05) during storage regardless of treatments. There were no significant differences (*p* > 0.05) in L*-values among the treatments at days 0 and 3 of storage. Tannic acid at 10 ppm showed the stronger effect on L*-value than other treatments: the breast meat added with 10 ppm tannic acid had the highest L*-value (lighter meat), and the control had the lowest value (darker meat) at Day 7. The meat with high lightness value is considered more acceptable by the consumer than the darker one because it can be considered old or spoiled [45]. Regardless of treatments, a*-values decreased during storage. However, no significant difference was found (*p* > 0.05) between treatments at day 0. This agreed with the results of Xiao *et al.* [39] who reported that a*- and L*-values of ground chicken meat decreased significantly after 7 days of refrigerated storage. Mancini and Hunt [6] reported that the decrease of a*-value during storage is due to the accumulation of metmyoglobin pigment. Tannic acid at 10 ppm showed a greater effect in preventing changes of a*-value than all other treatments but 5 ppm tannic acid after 3 days of storage. This is in agreement with the results of Maqsood and Benjakul [20] who found that all ground beef samples treated with tannic acid had higher oxymyoglobin and a*-value, and received a higher likeness score for color in the sensory evaluation. However, there was no significant difference in a*-value between tannic acid levels at 5 and 10 ppm tannic acid, and 5 ppm BHA during storage. In comparison, Luciano *et al.* [46] found that dietary tannins improved lamb minced-meat color stability and their shelf life. Changes in b*-values in chicken breast meat during storage were not significant even though tannic acid-treated meat showed higher values than control.

### 3.4. Volatiles Production

At day 0, the addition of more than 5 ppm tannic acid significantly reduced the amounts of pentane, heptane, pentanal and hexanal produced in the cooked chicken meat compared to the control. Addition of BHA also reduced the amounts of pentane, ethanol, pentanal and hexanal in the cooked meat (Table 4). Hexanal increased rapidly in cooked meat during storage due to a high degree of lipid oxidation. Many lipid oxidation-related aldehydes such as propanal, hexanal, and pentanal were also detected during storage of cooked meat. After 3 days of storage, tannic acid at 5 and 10 ppm showed the most significant effect on the formation of most of these volatiles (Table 5 and Table 6). For example, tannic acid at 10 ppm significantly decreased pentane, octane, hexanal, and pentanal formation during storage. Hexanal, which is considered as a good indicator for lipid oxidation [47,48], was significantly affected by 10 ppm of tannic acid at Day 7 of storage (Table 6). Heptanal and nonanal were formed and increased only in control and 2.5 ppm tannic acid-added samples (Table 5 and Table 6). Tannic acid at 10 ppm showed the strongest effect in preventing aldehydes’ formation in cooked meat. This was in agreement with and reflected the positive relationships between the aldehydes and the degree of lipid oxidation as reported by Nam *et al.* [49] (Table 1 and Table 6). Similar effects on the heptanal and nonanal formation were seen when 10 ppm of tannic acid was added to the cooked meat. Overall, the profile of volatiles indicated that tannic acid at 5 and 10 ppm effectively delayed the formation of lipid oxidation-related volatiles in cooked chicken breast meat during storage.

## 4. Conclusions

Tannic acid at 5 or 10 ppm could be effective in maintaining meat color and retarding lipid and protein oxidation, and off-odor-related volatile formation in ground chicken breast during storage. Therefore, tannic acid could be a good candidate as a natural antioxidant to prevent oxidative and color changes in raw and cooked chicken breast meat during storage.

## Figures and Tables

**Table 1:** 2-Thiobarbituric acid-reactive substances (TBARS) values of raw and cooked chicken breast meat with different levels of tannic acid during storage at 4 °C.

Time	Control	2.5 ppm	5 ppm	10 ppm	5 ppm	SEM
	(None)	Tannic	Tannic	Tannic	BHA	
*Raw meat*	TBARS (mg MDA/kg meat)					
Day 0	0.14 ^a,z^	0.13 ^a,z^	0.13 ^a,y^	0.12 ^a,x^	0.13 ^a,y^	0.01
Day 3	0.18 ^a,y^	0.17 ^a,y^	0.15 ^ab,x^	0.13 ^b,x^	0.18 ^a,x^	0.01
Day 7	0.34 ^a,x^	0.33 ^a,x^	0.17 ^bc,x^	0.14 ^c,x^	0.19 ^b,x^	0.01
SEM	0.00	0.01	0.01	0.01	0.02	
*Cooked meat*	TBARS (mg MDA/kg meat)					
Day 0	0.19 ^a,z^	0.15 ^b,y^	0.13 ^b,z^	0.11 ^bc,z^	0.14 ^b,y^	0.01
Day 3	1.42 ^a,y^	1.08 ^b,x^	0.57 ^c,y^	0.28 ^d,y^	0.97 ^b,x^	0.06
Day 7	2.23 ^a,x^	1.26 ^b,x^	0.79 ^c,x^	0.34 ^d,x^	0.99 ^c,x^	0.05
SEM	0.06	0.10	0.03	0.01	0.03	

^a–d^ Values with different letters within a row are significantly different (*p* < 0.05). *n* = 4; ^x–z^ Values with different letters within a column are significantly different (*p* < 0.05). Abbreviation: SEM, standard error of the mean; BHA, butylated hydroxyanisole; TBARS, 2-thiobabituric acid reactive substances; MDA, malondialdehyde.

**Table 2:** Protein oxidation of raw and cooked chicken breast meat with different levels of tannic acid during storage at 4 °C.

Time	Control	2.5 ppm	5 ppm	10 ppm	5 ppm	SEM
	(None)	Tannic	Tannic	Tannic	BHA	
*Raw meat*	Carbonyl content (nmole/mg protein)					
Day 0	0.54 ^a,y^	0.54 ^a,y^	0.52 ^a,y^	0.52 ^a,x^	0.53 ^a,y^	0.03
Day 3	0.72 ^a,x^	0.72 ^a,x^	0.70 ^a,x^	0.64 ^a,x^	0.71 ^a,x^	0.06
Day 7	0.82 ^a,x^	0.81 ^a,x^	0.77 ^a,x^	0.66 ^b,x^	0.77 ^a,x^	0.02
SEM	0.04	0.04	0.03	0.04	0.03	
*Cooked meat*	Carbonyl content (nmole/mg protein)					
Day 0	0.58 ^a,z^	0.57 ^a,y^	0.46 ^a,x^	0.45 ^a,y^	0.47 ^a,y^	0.07
Day 3	1.21 ^a,y^	1.13 ^a,xy^	0.62 ^b,x^	0.59 ^b,xy^	0.80 ^b,x^	0.05
Day 7	2.01 ^a,x^	1.38 ^b,x^	0.64 ^c,x^	0.60 ^c,x^	0.82 ^c,x^	0.10
SEM	0.03	0.14	0.08	0.04	0.03	

**^a–c^** Values with different letters within a row are significantly different (*p* < 0.05). *n* = 4; ^x–z^ Values with different letters within a column are significantly different (*p* < 0.05). Abbreviation: SEM, standard error of the mean; BHA, butylated hydroxyanisole.

**Table 3:** CIE color values of raw chicken breast meat with different levels of tannic acid during storage at 4 °C.

Time	Control	2.5 ppm	5 ppm	10 ppm	5 ppm	SEM
	(None)	Tannic	Tannic	Tannic	BHA	
L*-value						
Day 0	64.52 ^a,x^	64.28 ^a,x^	64.32 ^a,x^	64.40 ^a,x^	64.32 ^a,x^	0.17
Day 3	63.87 ^a,x^	63.74 ^a,x^	63.63 ^a,y^	64.04 ^a,x^	63.64 ^a,y^	0.14
Day 7	61.44 ^b,y^	62.45 ^ab,y^	62.58 ^a,z^	63.27 ^a,y^	62.44 ^ab,z^	0.24
SEM	0.29	0.16	0.14	0.18	0.08	
a*-value						
Day 0	8.53 ^a,x^	8.45 ^a,x^	8.43 ^a,x^	8.41 ^a,x^	8.43 ^a,x^	0.14
Day 3	6.50 ^b,y^	6.42 ^b,y^	6.71 ^ab,y^	7.00 ^a,y^	6.40 ^b,y^	0.10
Day 7	6.09 ^b,z^	6.07 ^b,y^	6.43 ^ab,y^	6.91 ^a,y^	6.35 ^ab,y^	0.15
SEM	0.09	0.20	0.09	0.12	0.13	
b*-value						
Day 0	20.09 ^b,x^	20.14 ^b,x^	19.63 ^b,x^	21.33 ^a,x^	20.21 ^b,x^	0.15
Day 3	19.03 ^c,y^	19.02 ^c,y^	19.75 ^bc,x^	21.41 ^a,x^	20.43 ^ab,x^	0.27
Day 7	20.34 ^ab,x^	20.26 ^ab,x^	19.88 ^b,x^	20.81 ^a,x^	20.53 ^ab,x^	0.20
SEM	0.19	0.18	0.29	0.21	0.16	

^a–c^ Values with different letters within a row are significantly different (*p* < 0.05). *n* = 4; ^x–z^ Values with different letters within a column are significantly different (*p* < 0.05). Abbreviation: SEM, standard error of the mean; BHA, butylated hydroxyanisole.

**Table 4:** Volatile profiles of cooked chicken breast meat with different level of tannic acid at day 0.

Compounds	Control	2.5 ppm	5 ppm	10 ppm	5 ppm	SEM
	(None)	Tannic	Tannic	Tannic	BHA	
	Total ion counts × 10^4^					
Pentane	238 ^a^	240 ^a^	0 ^b^	0 ^b^	0 ^b^	38
2-Propanone	5267 ^a^	3407	3325 ^a^	4171 ^a^	1923 ^a^	1057
Ethanol	8757 ^a^	8805 ^a^	8302 ^a^	8483 ^a^	3557 ^b^	830
2-Propanol	810 ^a^	551 ^a^	564 ^a^	494 ^a^	779 ^a^	142
Hexane	331 ^b^	277 ^b^	144 ^bc^	24 ^c^	830 ^a^	50
Heptane	56 ^ab^	50 ^b^	0 ^c^	0 ^c^	85 ^a^	7
Pentanal	566 ^a^	69 ^b^	0 ^b^	0 ^b^	0 ^b^	51
Octane	274 ^a^	148 ^ab^	143 ^ab^	54 ^b^	162 ^ab^	31
Hexanal	1761 ^a^	1447 ^a^	78 ^b^	0 ^b^	162 ^b^	174

^a–c^ Values with different letters within a row are significantly different (*p* < 0.05). *n* = 4. Abbreviation: SEM, standard error of the mean; BHA, butylated hydroxyanisole.

**Table 5:** Volatile profiles of cooked chicken breast meat with different levels of tannic acid at day 3.

Compounds	Control	2.5 ppm	5 ppm	10 ppm	5 ppm	SEM
	(None)	Tannic	Tannic	Tannic	BHA	
	Total ion counts × 10^4^					
Pentane	912 ^a^	766 ^a^	0 ^b^	0 ^b^	0 ^b^	50
Propanal	1459 ^a^	981 ^a^	0 ^b^	0 ^b^	0 ^b^	152
2-Propanone	3792 ^b^	2283 ^b^	5796 ^a^	5638 ^a^	2674 ^b^	403
Ethanol	5466 ^a^	6959 ^a^	4546 ^ab^	6540 ^a^	2845 ^b^	576
2-Propanol	4826 ^b^	8903 ^a^	8552 ^a^	4842 ^b^	5671 ^b^	461
Heptane	410 ^a^	303 ^a^	0 ^b^	0 ^b^	0 ^b^	31
Pentanal	3916 ^a^	1732 ^b^	0 ^c^	0 ^c^	110 ^c^	115
Octane	171 ^a^	60 ^b^	61 ^b^	0 ^b^	149 ^a^	17
Hexanal	11755 ^a^	9757 ^a^	257 ^b^	0 ^b^	924 ^b^	559
Heptanal	64 ^a^	56 ^a^	0 ^b^	0 ^b^	0 ^b^	3
Nonanal	77 ^a^	47 ^b^	0 ^c^	0 ^c^	0 ^c^	4

^a–c^ Values with different letters within a row are significantly different (*p* < 0.05). *n* = 4. Abbreviation: SEM, standard error of the mean; BHA, butylated hydroxyanisole.

**Table 6:** Volatile profiles of cooked chicken breast meat with different levels of tannic acid at day 7.

Compounds	Control	2.5 ppm	5 ppm	10 ppm	5 ppm	SEM
	(None)	Tannic	Tannic	Tannic	BHA	
	Total ion counts × 10^4^					
Pentane	1195 ^b^	2349 ^a^	0 ^c^	0 ^c^	0 ^c^	101
Propanal	350 ^a^	310 ^a^	0 ^b^	0 ^b^	0 ^b^	19
2-Propanone	6506 ^a^	4420 ^ab^	3682 ^ab^	3348 ^b^	3873 ^ab^	662
2-Propanol	9122 ^a^	1354 ^b^	1435 ^b^	1438 ^b^	1364 ^b^	249
1-Propanol	2625 ^a^	1228 ^b^	1377 ^b^	1440 ^b^	1322 ^b^	172
Heptane	302 ^a^	144 ^ab^	0 ^b^	0 ^b^	0 ^b^	42
Pentanal	3849 ^a^	4148 ^a^	0 ^b^	0 ^b^	126 ^b^	225
Octane	776 ^a^	407 ^b^	56 ^bc^	0 ^c^	200 ^bc^	83
Hexanal	67351 ^a^	51368 ^b^	621 ^c^	0 ^c^	1328 ^c^	537
Heptanal	252 ^a^	223 ^a^	0 ^b^	0 ^b^	0 ^b^	8
Nonanal	101 ^a^	76 ^ab^	0 ^b^	0 ^b^	0 ^b^	18

^a–c^ Values with different letters within a row are significantly different (*p* < 0.05). *n* = 4. Abbreviation: SEM, standard error of the mean; BHA, butylated hydroxyanisole.
